# Estimation of Hand Grip Strength Using a Grip Force Transducer in Healthy Volunteers

**DOI:** 10.7759/cureus.110278

**Published:** 2026-06-04

**Authors:** Ruchi Kothari, Archana Pawar, Sai Shanmukh Vemparala, Hariprasath Gopalakrishnan, Marina P Johny, Vineet Buradkar, Senthil Kumar, Swapnil P Chube, Prashanth A.

**Affiliations:** 1 Physiology, Mahatma Gandhi Institute of Medical Sciences, Wardha, IND; 2 Physiology, Anna Gowri Medical College and Hospital, Puttur, IND; 3 Physiology, Great Eastern Medical School and Hospital, Srikakulam, IND; 4 Physiology, Andhra Medical College, Visakhapatnam, IND; 5 Biochemistry, Madha Medical College and Research Institute, Chennai, IND; 6 Pediatrics, Government T.D. Medical College Hospital, Alappuzha, IND; 7 General Surgery, Holy Spirit Hospital and Research Centre, Mumbai, IND; 8 Physiology, Mahatma Gandhi Medical College and Research Institute, Chennai, IND; 9 Pharmacology, N.K.P. Salve Institute of Medical Sciences and Research Centre and Lata Mangeshkar Hospital, Nagpur, IND

**Keywords:** grip force transducer, hand grip strength, isometric exercise, normative data, surface electromyography

## Abstract

Background

Hand grip strength (HGS) is a vital measure of muscular function and serves a pivotal role in the identification of functional impairments, as well as in evaluating the effectiveness of therapeutic interventions in individuals with hand or upper extremity injuries undergoing rehabilitation. HGS represents a simple, objective, and reliable indicator of physical status, muscular performance, cardiovascular fitness, and functional disability.

The primary objective of the study was to estimate HGS using a grip force transducer and to establish normative reference data for a healthy population from central India. Secondary exploratory objectives included examining the relationships of HGS with anthropometric and physiological variables, such as age, body mass index (BMI), heart rate (HR), and electromyography (EMG) activity, to provide additional physiological context for interpreting grip strength performance.

Methods

The study was descriptive and cross-sectional in design and was conducted in the sports physiology laboratory of the Department of Physiology at a rural central Indian medical institute. A total of 400 volunteers aged 17-65 years were selected according to the inclusion criteria. HGS was assessed using a grip force transducer. Surface EMG measurements were obtained through disposable silver-silver chloride electrodes. Data acquisition was performed using the PowerLab system (ADInstruments Pty Ltd., Dunedin, New Zealand), and the analysis was carried out using LabChart Pro software (ADInstruments Pty Ltd., Dunedin, New Zealand).

Results

Of the 400 participants (220 men and 180 women), the mean age was 34 ± 11 years. The mean HGS was significantly lower in females than in their male counterparts, as revealed by the unpaired t-test. There was also a significant inverse correlation between mean HGS and age in males (r = -0.30) and females (r = -0.36). Although HR was significantly positively correlated with mean HGS, the correlation coefficients were relatively weak (for males, r = 0.23, and for females, r = 0.18), indicating that HR explains only a small proportion of the variability in HGS. The root mean square values of EMG demonstrated a statistically non-significant, weak positive correlation with mean HGS.

Conclusion

The present research has established age-specific normative values of HGS, estimated using a grip force transducer, for the healthy population of central India. A statistically significant difference in grip strength was noted between the sexes, with men exhibiting greater grip strength than women. Age and HGS were also found to be inversely related, whereas a statistically significant positive correlation was observed between HR and HGS.

## Introduction

The hand is considered to be the most advanced and highly specialized example of the musculoskeletal system in humans. Its intricate anatomical and neuromuscular organization allows it to execute even complex tasks with precision [1]. Hand grip is an important indicator of upper extremity function that enables humans to perform a wide range of activities requiring strength and dexterity. In medical terms, grip is defined as a powerful dynamic action involving the flexion of all finger joints, as well as the thumb being utilized as a counterbalancing agent for the object to be grasped between the palm and the fingers [2]. This coordinated movement is crucial for performing various tasks that require strength and precision.

Hand grip strength (HGS) is a significant index of muscular performance and an essential measure for both clinical and research settings. Grip strength is gauged by estimating the quantum of immovable force which the hand uses to compress a force transducer, and it serves as a reliable and valid objective index of the integral functionality of the upper extremity [3]. It reflects an individual’s musculoskeletal health, functional quotient, and potential for rehabilitation. Assessing HGS is crucial for evaluating upper limb impairment. It helps determine the initial deficit in hand muscle power, establish treatment goals, gauge improvement during rehabilitation, provide evidence for the efficacy of different treatment plans, and assess a patient's readiness to return to work [4]. In addition to these applications, creating awareness regarding the significance of one’s own HGS would also help address the growing crisis that most developing countries are confronting nowadays. The serious challenge of rising obesity rates has arisen due to reduced physical activity and underutilization of muscle power owing to a sedentary lifestyle [5].

Although a cluster of reference ranges for HGS classified on the basis of age, gender, and dominance from different ethnicities exists, and diverse measurement modalities have been employed [6-10], such normative data are applicable only to the particular region from which the data originate. Using norms from Western literature would be inappropriate from an Indian perspective owing to genetic, nutritional, demographic, and environmental factors that may impact HGS. Population-specific reference standards for grip strength measurements are essential to ensure accurate interpretation and evaluation of strength outcomes for the identification of grip-strength impairments.

Further, HGS is increasingly recognized as a simple surrogate marker of overall muscle strength, nutritional status, physical fitness, and future health outcomes. In addition to establishing normative values, understanding the relationship of HGS with anthropometric and physiological indicators such as body mass index (BMI), A Body Shape Index (ABSI), heart rate (HR), and electromyographic (EMG) activity may provide insights into factors influencing muscular performance. ABSI is a marker of central adiposity and body composition that has been associated with cardiometabolic risk. HR was included as an indicator of cardiovascular and autonomic response during muscular activity, whereas surface EMG was used to assess muscle activation patterns associated with grip force generation. Examining these parameters alongside HGS may improve understanding of physiological factors and body shape characteristics contributing to muscular performance.

Hence, this study was conceived with the primary objective of estimating HGS using a grip force transducer and generating reference values in a central Indian population aged 17-65 years. By focusing on a representative sample of healthy individuals, this research establishes baseline data essential for comparative analysis in various medical conditions and rehabilitation outcomes. The secondary objectives of the study were to evaluate the association of HGS with age, BMI, ABSI, HR, and EMG activity and to explore physiological and anthropometric factors that may influence grip strength performance, since limited data are available in this area. 

## Materials and methods

Study design and setting

This descriptive observational study was conducted in the sports physiology laboratory of the Department of Physiology of a rural medical college in central India. A total of 400 subjects were enrolled and selected via the convenience sampling technique. All participants provided signed written informed consent before being enrolled in the study. Approval of the Institutional Ethics Committee was duly obtained as per Reference No. MGIMS/IEC/ANAT/17/2019 dated 05/01/2019. OpenEpi, Version 3.01 (Centers for Disease Control and Prevention, Atlanta, GA, USA), was utilized to calculate the sample size. Assuming an effect size of 0.6, alpha error of 0.05, and power of 80%, the software yielded a minimum required sample size of 383. Taking into account a 10% non-response and refusal rate, a total of 400 subjects were enrolled in the study.

Selection criteria

Participants aged 17-65 years, willing to provide informed consent, able to take part in all data collection procedures, and free from neuropathic or musculoskeletal conditions that could limit performance in physical assessment were included. Since HGS investigation is an indicator of autonomic function, individuals suffering from any disorders that may alter autonomic reflexes were not recruited. To ensure inclusion of healthy participants, all volunteers underwent a detailed screening process prior to enrolment. This included a structured medical history interview to identify previously diagnosed hypertension, diabetes mellitus, ischemic heart disease, cardiac arrhythmias, nephropathy, psychiatric illness, and any family history of metabolic disorders such as diabetes or hypertension; assessment of lifestyle habits, including smoking status, through self-reporting; and baseline clinical evaluation, including measurement of blood pressure (BP) and HR before testing. Only healthy individuals fulfilling the inclusion criteria and without any history suggestive of autonomic dysfunction were included in the study.

Data collection and measurement of variables

Anthropometrics 

The anthropometric data of all subjects were collected. Age was noted in years and months. Standing height was measured without footwear in centimeters, along with weight in kilograms, using a Phoenix Height Weight Body Mass Index machine (Model: PBMI-200; Phoenix, New Delhi, India), which automatically displayed BMI. Waist circumference (WC) (in centimeters) was measured using standard procedures [11] on bare skin in the standing posture. ABSI, an anthropometric measure calculated from WC, was computed according to the formula [12,13]: ${ABSI} = \frac{\mathrm{WC}}{\mathrm{BMI}^{2/3} \times \mathrm{Height}^{1/2}}$.

Hand Grip Strength (HGS) and Electromyography (EMG)

Participants were familiarized with the testing procedure before data acquisition. Hand dominance was determined by self-report, with participants being asked which hand they routinely used for writing and performing skilled daily activities. Grip strength measurements were obtained from the dominant hand, and the normative reference values presented in the study correspond to the dominant hand. HGS, expressed in Newton (N), was recorded following the standard protocol [14] using a grip force transducer (MLT004/ST, ADInstruments Pty Ltd., Dunedin, New Zealand), which was calibrated according to the manufacturer's recommendations before commencement of data collection sessions. Calibration and functionality checks were performed periodically throughout the study period to ensure consistency of measurements.

The subject was seated comfortably on a chair, and the dominant forearm was exposed. The shoulder was adducted and neutrally rotated, the elbow flexed at 90°, the forearm in a neutral position, and the wrist extended from 0° to 15°, with the forearm supinated and resting comfortably on an arm support. Participants were instructed to avoid compensatory movements of the shoulder or trunk during testing. The surface electrodes, namely red (positive) and white (negative), were applied over the proximal forearm on the belly of the muscle, while the ground electrode was placed on the anterior distal forearm. A set of two attempts, with a gap of 20 seconds, was given to each participant to record maximum grip strength. Maximum HGS, corresponding to 100% of grip force, and the best of the two attempts were considered for analysis. An average HGS was then calculated using LabChart Pro software by sampling the entire recording duration [15]. Data acquisition lasted approximately three to five minutes. For quantitative analysis, a representative 20-second segment free from movement artifacts and signal disturbances was selected by the software. The mean grip strength value reported in the study was calculated from this standardized analysis window.

Systolic and diastolic BP, along with HR, were recorded both prior to and after testing. Surface electromyography is a non-invasive method of recording electrical activity during voluntary muscle contraction. When a subject squeezes the transducer, force is transmitted to it and recorded by the data acquisition system and amplified later. EMG signals were amplified by a Bio Amplifier (FE132; ADInstruments Pty Ltd.) after applying silver-silver chloride disposable electrodes over the forearm. The root mean square (RMS) values, expressed in millivolts (mV), enabled quantification of muscle activation calculated from the raw EMG signal. A band-pass filter between 25 and 400 Hz was used to process the raw EMG signal. RMS was sampled at a rate of 1000 Hz. Due to the varied morphology of raw EMG potentials, a triangular smoothing window of 0.5 seconds was used [15].

Statistical analysis

Data were statistically analyzed using IBM SPSS Statistics for Windows, Version 26 (Released 2013; IBM Corp., Armonk, NY, USA). To assess normality, continuous variables following a normal distribution were expressed as mean ± standard deviation (SD) and compared between groups using the Student t-test. Categorical variables were summarized using the chi-square (χ²) test. Pearson’s correlation analysis was performed to evaluate linear relationships between continuous variables. Statistical significance was defined as a two-tailed p-value of < 0.05.

## Results

The descriptive statistics of 220 males and 180 females in the age range of 17 to 65 years are presented in Table 1. The difference in mean age between the sexes was statistically non-significant (t = 0.65, p = 0.51), whereas the other demographic parameters showed a significant difference between males and females.

**Table 1:** Descriptive statistics of males and females Max HGS: Maximum hand grip strength; N: Newton; RMS: Root mean square; EMG: Electromyography; mV: millivolts; HR: Heart rate; BMI: Body mass index; Kg/m^2^: Kilograms per square meter; ABSI: A body shape index; SD: Standard deviation

Parameters	Males (n = 220)			Females (n = 180)		
	Minimum	Maximum	Mean ± SD	Minimum	Maximum	Mean ± SD
Age (years)	17.00	65.00	35.56 ± 11.05	17.00	65.00	36.23 ± 9.00
Max HGS (N)	254.66	446.64	350.65 ± 95.99	165.79	271.91	218.85 ± 53.06
RMS of EMG (mV)	0.14	0.48	0.31 ± 0.17	0.10	0.34	0.22 ± 0.11
HR (beats per minute)	68.00	106.00	87 ± 19.00	76.00	92.00	84 ± 8.00
BMI (kg/m^2^)	18.36	25.70	22.03 ± 3.67	19.03	28.07	23.55 ± 4.52
ABSI	0.44	0.95	0.69 ± 0.26	0.47	0.85	0.66 ± 0.19

**Figure 1:**
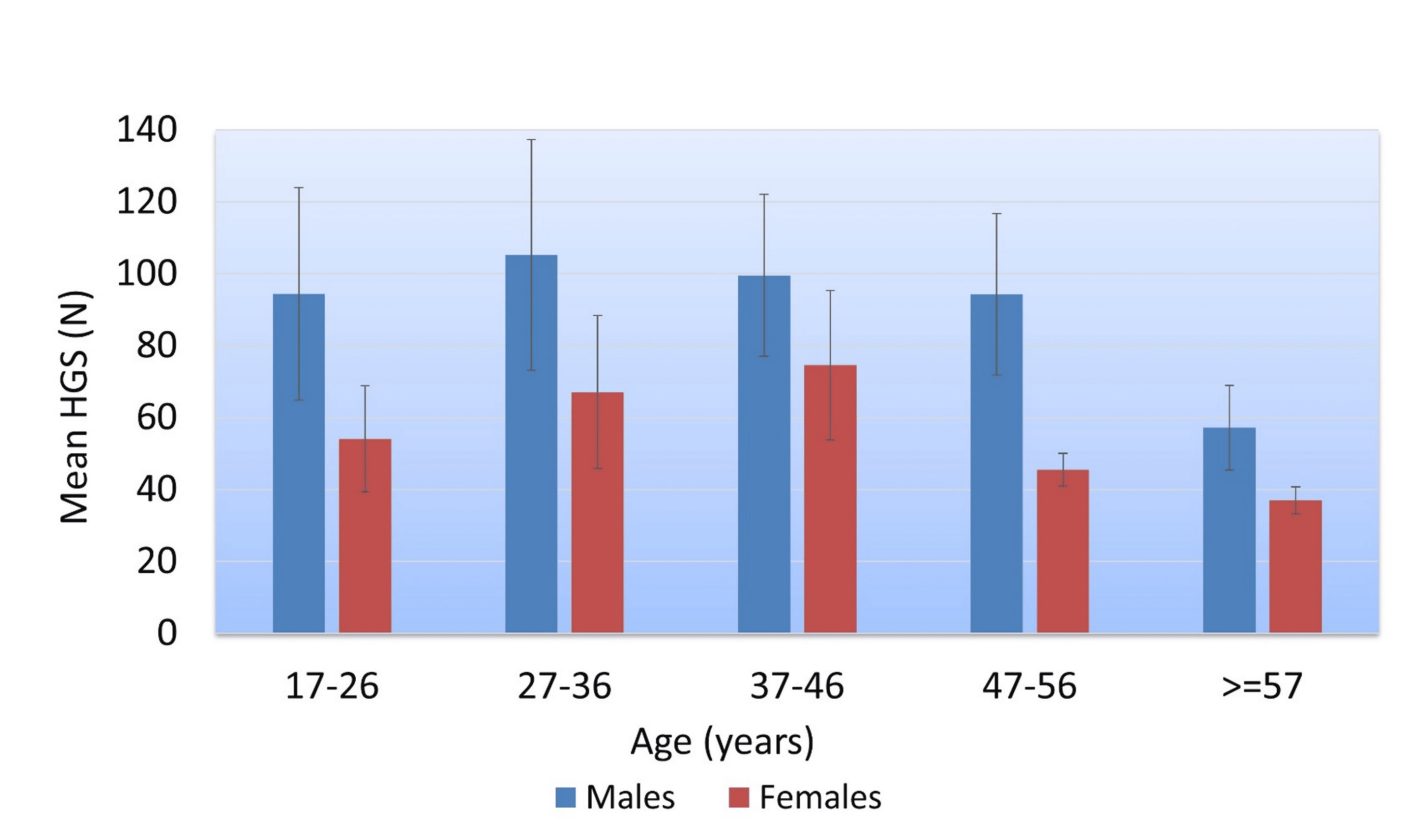
Mean hand grip strength of dominant hand in males and females

The study subjects were categorized sex-wise as males and females in the age groups of 17-26 years, 27-36 years, 37-46 years, 47-56 years, and >57 years. The mean and SD values of grip strength of the dominant hand in both sexes across different age groups are depicted in Table 2 and illustrated in Figure 1. The maximum mean HGS in males was seen in the age group 27-36 years, whereas in females, the highest HGS values were observed in the age group 37-46 years.

**Table 2:** Hand grip strength values of dominant hand in males and females SD: Standard deviation; HGS: Hand grip strength

Age group (years)	Dominant hand grip strength (males)			Dominant hand grip strength (females)		
	Mean ± SD HGS (N)	95% confidence interval for mean		Mean ± SD HGS (N)	95% confidence interval for mean	
		Lower bound	Upper bound		Lower bound	Upper bound
17-26	94.40 ± 29.53	87.95	100.84	54.06 ± 14.77	50.53	57.58
27-36	105.23 ± 32.05	97.02	113.43	67.04 ± 21.29	61.44	72.63
37-46	99.53 ± 22.51	87.33	101.18	74.57 ± 20.81	66.79	82.34
47-56	94.26 ± 22.51	87.33	101.18	45.49 ± 4.57	42.85	48.12
≥57	57.18 ± 11.73	49.72	64.63	36.99 ± 3.71	33.88	40.09

Upon statistical analysis using the unpaired t-test, the mean and SD hand grip strength values in females were found to be significantly lower than those of males in all age groups. The difference was statistically significant (p < 0.001), as shown by the t-statistic values in Table 3.

**Table 3:** Comparative analysis of mean hand grip strength of males and females All p-values were evaluated using the Student t-test between the two sexes. SD: Standard deviation

Age group (years)	Dominant hand grip strength (N) (males)	Dominant hand grip strength (N) (females)	Statistics	
	Mean ± SD	Mean ± SD	t	p-value
17-26	94.40 ± 29.53	54.06 ± 14.77	16.69	<0.001
27-36	105.23 ± 32.05	67.04 ± 21.29	13.70	<0.001
37-46	99.53 ± 22.51	74.57 ± 20.81	11.41	<0.001
47-56	94.26 ± 23.67	45.49 ± 14.57	24.18	<0.001
≥57	57.18 ± 11.73	36.99 ± 13.71	15.86	<0.001

The correlations among age, mean HGS, RMS, HR, and BMI using Pearson’s correlation coefficient are presented in Table 4. The table illustrates a statistically significant (p < 0.001) inverse correlation between age and mean HGS in both males and females. The RMS of EMG and mean HGS were positively correlated, but the relationship was not statistically significant. A weak positive correlation was observed between HR and mean HGS, which was statistically significant. It is evident that BMI with mean HGS and BMI with RMS showed no significant correlation in both males and females.

**Table 4:** Correlation analysis of study parameters between the two sexes All the r-values reported are based on Pearson’s correlation test. A p-value < 0.05 is considered statistically significant. HGS: Hand grip strength; RMS: Root mean square; HR: Heart rate; BMI: Body mass index; S: Significant; NS: Non-significant, r: Pearson's correlation coefficient

Correlation between	Males		Females	
	r-value	p-value	r-value	p-value
Age and mean HGS	-0.30	p < 0.001 (S)	-0.36	p < 0.001 (S)
RMS and mean HGS	0.03	0.656 (NS)	0.01	0.893 (NS)
HR and mean HGS	0.23	p < 0.001 (S)	0.18	0.015 (S)
BMI and mean HGS	0.05	0.445 (NS)	0.02	0.789 (NS)
BMI and RMS	-0.01	0.882 (NS)	-0.04	0.592 (NS)

## Discussion

Grip strength estimation carries immense importance in ascertaining impairments, determining goals, and confirming the efficacy of therapeutic plans for patients with upper extremity injuries. Normative data of HGS have been referenced for the healthy central Indian population in this study, which should be considered representative of the study population and regional setting rather than the entire Indian population. It was found that HGS gradually increased with age, peaking in the 27-36 years age group in males and 37-46 years in females. A gradual decline was seen with advancing age, with a minimum observed in the 57-65 years age group in both sexes. There was a curvilinear relationship between age and HGS, which corroborates the findings of Walankar et al. [6], who established normative ranges for HGS using a Jamar hand dynamometer in individuals aged 21-80 years. In their study, the maximum HGS was found in the 31-40 years age group, followed by a gradual decrease with advancing age, reaching a minimum in the 71-80 years age group. The age-related decline in muscle strength was attributed to multiple factors, including a reduction in the number of muscle fibers and alterations in fiber morphology, particularly atrophy of fast-twitch (Type II) fibers.

A higher HGS in males was noted in the current study compared to females, which is in agreement with the studies of Angst et al. [1] and Walankar et al. [6], who also demonstrated gender to be one of the most crucial determinants of HGS. Angst et al. [1], studying 978 volunteers aged 18-85 years, and Walankar et al. [6], investigating 600 volunteers aged 21-80 years, consistently reported that males generally have stronger HGS than females. The higher grip strength observed in males is consistent with previous studies and is generally attributed to differences in muscle mass, hormonal influences, and anthropometric characteristics [16].

In our study, it was found that HGS was higher in subjects with normal weight and lowest in overweight individuals in both sexes, although the difference did not reach statistical significance. Ravisankar et al. [17] and Prashanth et al. [18] have also documented similar observations. Aligning with Ravisankar et al., BMI in males and females in the present study showed a weak positive correlation with HGS, which was not statistically significant. Similar observations were reported by Kothari et al. [19], who demonstrated a positive correlation between BMI and HGS among the older population. Lad et al. conducted a study on 180 subjects - 90 boys and 90 girls aged 18-21 years - and reported a negative correlation between BMI, body fat percentage, and hand grip endurance; however, this was not significant in the overweight subgroup [20]. HGS was also evaluated by Cem et al. in a study on 124 males and 77 females aged 18-29 years, and it was observed that HGS was positively correlated with BMI and age [21]. Hence, according to the existing literature, it is evident that HGS is significantly influenced by age, sex, and BMI.

The observed statistically significant positive correlation between HR and HGS in our study may reflect shared influences of general physical fitness, underlying autonomic responsiveness, and cardiovascular adaptations associated with greater muscular fitness. Individuals with better muscle performance may exhibit enhanced sympathetic responsiveness during voluntary isometric contraction, resulting in a more pronounced cardiovascular response. Furthermore, greater muscle mass and neuromuscular activation may contribute to both stronger grip performance and increased cardiac output demands during testing. Nevertheless, the observed correlation was modest, and causality cannot be inferred from the cross-sectional design. Also, since the magnitude of the association was weak, the finding should be interpreted cautiously, as it does not necessarily indicate a strong physiological relationship. Variability in grip strength may also be influenced by unmeasured factors, including habitual physical activity, occupational demands, and nutritional status, which should be considered in future research.

One of the parameters investigated in this study was the RMS of the EMG signal, which could reflect skeletal muscle activity during the performance of isometric exercise in real time. In our study, EMG signals were positively associated with muscle strength, indicating better electrical activity of muscles in individuals with greater grip strength. However, since the association was not statistically significant, the present findings do not provide sufficient evidence to establish a meaningful relationship between EMG activity and grip strength in this population. 

Limitations of the study

This study possesses some limitations that should be considered while interpreting the findings. The study relied solely on healthy volunteers recruited using convenience sampling. Therefore, the findings may not be fully representative of the ethnic, socioeconomic, nutritional, and geographical diversity of the entire Indian population, so caution should be exercised while extrapolating the normative values to other demographic groups. As this was a single-center study conducted in rural central India, the generated reference values should be interpreted primarily as regional normative data. Further, percentile-based normative values would have provided clinically useful cut-off points for identifying individuals with reduced grip strength, but the subgroup sample sizes in the present study may not be sufficient to generate stable population-level percentile estimates across all age and sex categories. Although age-stratified analyses were performed, the use of relatively broad age categories may have masked more gradual age-related changes in grip strength. Future multicentric studies involving participants from different parts of India, with narrower age intervals or continuous age-based modeling, are warranted to establish nationally representative percentile-based reference standards.

Many factors known to influence HGS were not formally quantified or adjusted for in the analysis. These include habitual physical activity levels, occupational workload, dietary and nutritional status, socioeconomic characteristics, and participation in sports or resistance training. The absence of these measurements may have introduced residual confounding and may partly explain inter-individual variability in grip strength. The distribution of right- and left-hand dominant participants was not separately analyzed, and potential differences related to hand dominance could not be explored in detail.

Grip strength measurement at a single time point may not capture alterations occurring over time or account for factors such as fatigue or diurnal variability in HGS. To minimize variability and maintain consistency and validity, all HGS measurements were performed using the same grip force transducer connected to the same PowerLab data acquisition system throughout the study period. The equipment was checked for proper functioning and standardized before data collection sessions as per manufacturer recommendations. In addition, all recordings were conducted in the same laboratory setting using a uniform protocol for participant positioning, instructions, duration of contraction, and data acquisition. To further reduce interobserver and procedural variability, the measurements were obtained by trained investigators under supervised laboratory conditions. The same software was used uniformly for the analysis of all recordings. Since a single calibrated instrument and standardized methodology were employed for all participants, the possibility of measurement inconsistency affecting the normative data was minimized.

## Conclusions

The present study established age- and gender-specific normative values for HGS in a healthy central Indian population using a grip force transducer. HGS was significantly higher in males compared to females and demonstrated a decline with advancing age in both sexes. Although a positive trend was observed between RMS values of EMG and HGS, the association was not statistically significant; therefore, no definitive conclusion regarding the relationship between muscle electrical activity and grip strength can be drawn from the present data. The absence of significant associations between BMI, ABSI, and HGS suggests that central adiposity indicators alone may not adequately predict muscle strength in healthy adults, so they should not be used as substitutes for direct assessment of muscular fitness. Conversely, the observed positive association between HR and HGS may reflect underlying physiological interactions between muscular performance and cardiovascular responsiveness. The study findings provide preliminary reference data to aid clinical evaluation, contributing to rehabilitation monitoring and therapeutic strategies in clinical practice.
